# Effects of influent C/N ratios and treatment technologies on integral biogas upgrading and pollutants removal from synthetic domestic sewage

**DOI:** 10.1038/s41598-017-11207-y

**Published:** 2017-09-07

**Authors:** Jie Xu, Xue Wang, Shiqing Sun, Yongjun Zhao, Changwei Hu

**Affiliations:** 1 College of Biological Chemical Science and Engineering, Jiaxing University, Jiaxing, 314001 P.R. China; 2 Shanghai Public Green Space Construction Affairs Center, Shanghai, 201100 China

## Abstract

Three different treatment technologies, namely mono-algae culture, algal-bacterial culture, and algal-fungal culture, were applied to remove pollutants form synthetic domestic sewage and to remove CO_2_ from biogas in a photobioreactor. The effects of different initial influent C/N ratios on microalgal growth rates and pollutants removal efficiencies by the three microalgal cultures were investigated. The best biogas upgrading and synthetic domestic sewage pollutants removal effect was achieved in the algal-fungal system at the influent C/N ratio of 5:1. At the influent C/N ratio of 5:1, the algal-fungal system achieved the highest mean chemical oxygen demand (COD) removal efficiency of 81.92% and total phosphorus (TP) removal efficiency of 81.52%, respectively, while the algal-bacterial system demonstrated the highest mean total nitrogen (TN) removal efficiency of 82.28%. The average CH_4_ concentration in upgraded biogas and the removal efficiencies of COD, TN, and TP were 93.25 ± 3.84% (v/v), 80.23 ± 3.92%, 75.85 ± 6.61%, and 78.41 ± 3.98%, respectively. These results will provide a reference for wastewater purification ad biogas upgrading with microalgae based technology.

## Introduction

In rural areas of China, one of the main water pollution sources is domestic sewage, which contains abundant carbon, nitrogen, and phosphorus compounds. Recently, more than 90% of the domestic sewage in rural areas was directly discharged into natural waters without appropriate treatment, eventually causing water eutrophication^1^. Many studies indicated that conventional water treatment processes, including oxidation ditch processes^2^, anaerobic-anoxic-axic (A^2^/O)^3^, University of Cape Town (UCT)^4^, Bardenpho, and sequencing batch reactor (SBR)^5^, achieved moderate success in removing pollutants. However, these processes generally entail enormous land requirements, operational costs, complex operations, and large volumes of waste sludge production and are therefore not practicable in rural areas in China.

Conventional activated sludge systems will consume around half of the whole energy to convert chemical oxygen demand (COD) into the greenhouse gas CO_2_. The pollutants (i.e. carbon, nitrogen and phosphorus sources) in wastewater can be assimilated by microalgal species as nutrients for heterotrophic or mixotrophic growth^6^. Hence, as an alternative for the purification of domestic sewage, biological wastewater treatment system using microalgae is currently attracting increased interest because of its low construction and maintenance costs, minimal energy consumption, freedom from spatial restriction during operation, as well as high removal efficiency^7^. Study has shown that 85–88% of COD, 78–83% of total nitrogen (TN), and 73–80% of total phosphorus (TP) could be removed from wastewater by cultivating the algae *Chlorella* sp.^8^. Kumar *et al*. (2010) reported that treating digested piggery effluent with *Chlorella vulgaris* (*C. vulgaris*.) achieved removal efficiencies of 100% for TP and 78% for NH_4_^+^-N^9^. In addition, culturing certain species of microalgae with sanitary sewage might enable the harvest of potentially high added value microalgae biomass and the metabolic products, such as proteins and fatty acids^10, 11^. Therefore, microalgae-based technology is suitable for wastewater treatment because of its high effectiveness and low-cost^12, 13^.

In previous studies, significant improvement in the removal of xenobiotics from wastewaters has been observed with algae-bacteria/fungus–algae co-cultivation^14, 15^. The success of this technology is dependent on a mutually beneficial relationship, that is, by fixing CO_2_ which is produced by fungi/bacteria, algae can synthesize carbon source in the form of sugars and nutrients for fungi/bacteria through photosynthesis^16^. Therefore, pretreating wastewater by algal-fungal culture or algal-bacterial culture has an advantage on higher pollutants removal efficiencies comparing with mono-cultivation of algae cells, thereby offering a promising and efficient way to treat wastewater.

Biogas represents a renewable energy source based on its high CH_4_ content. Raw biogas usually consists of methane (47–65%), carbon dioxide (34–41%), and other trace compositions including hydrogen sulfide, water vapor, etc.^17^. However, the CH_4_ content of more than 90% (v/v) in biogas is required for vehicle fuel according to the natural gas standard^7^. The relatively high content of CO_2_ in raw biogas will lower its heat content as well as increase its energy demand for compression and transportation. Therefore, CO_2_ removal is essential for biogas upgrading. Numerous methods, such as physical absorption, chemical conversion, membrane separation, pressure swing adsorption, and cryogenic separation, have been used to upgrade biogas^17^. However, these methods require large amounts of energy, auxiliary materials, and chemicals, as well as generate wastes that require further treatment^18^. Moreover, the CO_2_ separated from the raw biogas using the above-mentioned methods is usually released directly into the atmosphere and result in greenhouse gas emission. Among the various strategies for biogas upgrading by CO_2_ removal, the biological sequestration of CO_2_ using photosynthetic microalgae has been receiving considerable attention because of the relatively high CO_2_ fixation capability of microalgae^11^. There are a limited number of studies on the integration of digestate treatment procedures and biogas upgrading via microalgae culture systems^8^. Accordingly, using microalgal growth to remove CO_2_ from crude biogas is an economic and efficient method for biogas upgrading, which makes biogas upgrading by microalgal culturing an economically convenient technique when used in conjunction with wastewater treatment^8, 19^.

Microalgal biomass production exhibits potentials for both pollutants reduction and CO_2_ removal. However, microalgal growth is also affected by organic matter and nutrient concentrations in wastewater as well as the presence of other heterotrophic microorganisms^20^. Fungi and bacteria can also have strong degradation abilities for certain wastewater contaminants and can be associated with microalgae to form immobilization systems of algal-fungal culture or algal-bacterial culture with multiple functions. The advantages of these associations in wastewater treatment are: (i) improved collection of biomass from wastewaters, (ii) easily recycled and manipulated consortia, (iii) improved features of microalgae such as thermal stability and productivity, and (iv) harvestable bioresource from proliferated microalgae biomass^19, 21, 22^. The use of biogas and sewage as raw materials can not only output high-grade biogas through the microalgae photosynthesis to assimilate carbon dioxide, but also can purify sewage by accumulating C, N, and P in sewage with microalgae. When compared with the traditional biogas upgrading and sewage purification technology, environmental friendly and cost-effective are the biggest advantages. Some researches on biogas upgrading or sewage purification resulted in methane loss and energy consumption, or lead to the increase of effluent^18^. Microalgae-based technology was therefore developed for simultaneously treating wastewater and upgrading biogas in recently years. Combination of wastewater treatment and biogas upgrading is indispensable because of its highly economically convenient comparing with wastewater treatment by using microalgae only^23^. Besides, the high cost generated from microalgae biomass harvesting is one of the major bottlenecks for the industrialization of algae-based technology because of the small microalgal size, negative surface charge, and low biomass concentration of microalgae^24^. Co-culture of microalgae with fungi/bacteria can well solve this problem. The bio-flocculation based microalgae assisted with fungi/bacteria is regarded as one of the best ways to realize the large-scale separation and microalgae recovery^25^. The energy consumption and the cost of the subsequent enrichment process have been significantly reduced^14^. At present, the few studies on couple biogas upgrading with wastewater treatment in photobioreactors provide little information on the influence of operational conditions on strain composition. Influent C/N ratio is the main factor affecting biological wastewater treatment processes^26^. However, thus far, only a few studies have evaluated the effects of different influent C/N ratios on the efficiency of domestic sewage treatment by microalgae^27–29^. Overlow or overhigh C/N ratios in the influent may result in low growth rate of microalgae and low removal efficiencies of nitrogen and phosphate in wastewater. In addition, the C/N ratio is also vital to the growth of microalgae when cocultivation with fungi or bacteria.

This study proposes an integrated approach for synthetic domestic sewage treatment and biogas upgrading through three algal culture methods (mono-algal culture, algal-fungal culture, and algal-bacterial culture). The purposes of this study were to (1) evaluate the effects of three different microalgae culture methods on sewage purification and biogas upgrading; and (2) identify the optimal influent C/N ratio to achieve the highest efficiencies of pollutants removal combined with biogas upgrading in synthetic domestic sewage.

## Results and Discussion

### Physicochemical variations

Changes in values of the sewage pH and DO are shown in Table 1. These changes were similar under all tested influent C/N ratios. The pH ranged from 7.11 to 7.54 in the influent, which were optimum values for the microalgae cultivation and the prevention of ammonia toxicity and phosphate precipitation^30^. No significant difference (*p* > 0.05) was observed among the three algae source cultures for pH values in the influent or effluent. However, for all treatments, pH of the influent was significantly higher (*p* < 0.05) than that in the effluent, which was typically below pH 8.0 (Table 1). The pH values observed in this study were consistent with those found by Papazi *et al*. (2008), who reported that pH values slightly varied between 6.0 and 7.5 under CO_2_ 30% (v/v) with *Chlorella minutissima* ^31^. This phenomenon meant that the biogas CO_2_ content in this research met the requirement for microalgae growth. Therefore, the CO_2_ consumption of the suspension culture only slightly affected the pH, which was rather related to the removal of organic pollutants. For instance, the reduced pH partly depends on the extent of nitrification in the photobioreactor, since ammonium volatilization processes play a negligible role in TN removal^13^.

**Table 1:** Means ± standard deviations of the physico-chemical parameters of influent and effluent for domestic sewage of three different methods of treatments at different influent C/N ratio.

C/N ratio		Influent		Effluent	
		pH	Do (mg L^−1^)	pH	Do (mg L^−1^)
**Mono-algae culture**					
C2.5N1-COD100	Low COD level	7.42 ± 0.23	7.94 ± 2.03	6.45 ± 0.31	6.37 ± 1.92
C5N1-COD200	Medium COD level	7.31 ± 0.19	7.42 ± 1.85	6.11 ± 0.16	6.52 ± 1.41
C10N1-COD400	High COD level	7.19 ± 0.11	2.91 ± 1.07	6.32 ± 0.14	1.95 ± 1.13
C10N1-TN20	Low TN level	7.28 ± 0.25	8.11 ± 1.94	7.03 ± 0.11	6.14 ± 1.02
C5N1-TN40	Medium TN level	7.51 ± 0.28	7.33 ± 1.56	7.12 ± 0.19	6.43 ± 1.27
C2.5N1-TN80	High TN level	7.26 ± 0.13	6.98 ± 1.39	6.39 ± 0.24	5.78 ± 0.82
**Algal-fungal culture**					
C2.5N1-COD100	Low COD level	7.44 ± 0.17	8.12 ± 2.11	6.87 ± 0.31	6.09 ± 1.17
C5N1-COD200	Medium COD level	7.11 ± 0.26	7.63 ± 1.55	6.93 ± 0.15	6.28 ± 1.56
C10N1-COD400	High COD level	7.52 ± 0.15	2.54 ± 1.18	7.04 ± 0.19	2.17 ± 1.08
C10N1-TN20	Low TN level	7.47 ± 0.19	6.91 ± 1.94	6.39 ± 0.21	6.32 ± 1.26
C5N1-TN40	Medium TN level	7.33 ± 0.21	7.72 ± 1.83	6.57 ± 0.16	6.71 ± 1.34
C2.5N1-TN80	High TN level	7.35 ± 0.32	6.53 ± 0.97	6.41 ± 0.18	6.03 ± 1.27
**Algal-bacterial culture**					
C2.5N1-COD100	Low COD level	7.54 ± 0.27	8.51 ± 1.84	6.58 ± 0.16	6.92 ± 1.57
C5N1-COD200	Medium COD level	7.41 ± 0.18	7.93 ± 0.92	6.77 ± 0.18	6.31 ± 1.25
C10N1-COD400	High COD level	7.48 ± 0.29	2.39 ± 1.46	6.32 ± 0.14	1.84 ± 0.93
C10N1-TN20	Low TN level	7.34 ± 0.15	6.77 ± 1.01	6.21 ± 0.17	6.04 ± 1.16
C5N1-TN40	Medium TN level	7.53 ± 0.24	7.02 ± 0.87	6.49 ± 0.16	6.23 ± 1.28
C2.5N1-TN80	High TN level	7.41 ± 0.12	6.15 ± 0.59	6.63 ± 0.26	5.93 ± 1.29

The DO of the wastewater slightly decreased during the experimental period. The biggest decrease varied from 8.12 ± 2.11 mg L^−1^ to 6.09 ± 1.17 mg L^−1^ in the C2.5N1-COD100 treatment by algal-fungal culture. However, this variation ranged below the excessive DO level (35 mg L^−1^, thereby would not inhibit microbial growth^32^. Carvalho *et al*. (2006) have reported that in a closed photobioreactor (similar to the photobioreactor used in this study), O_2_ in the influent might inhibit microalgal growth because of photorespiration^33^. This phenomenon did not occur in this research because of relatively high initial CO_2_ concentrations (28.43 ± 3.04%, v/v) in the photobioreactor and high organic carbon values in the influent^34^.

### Growth of the three selected cultures at various influent C/N ratios

Biomass productivity is a key parameter to analyze the potential of the three selected cultures to remove CO_2_. It generally varies with operational factors, such as light intensity, pH, working volume of the photobioreactor, and initial CO_2_ concentration in the simulated biogas^20^. Studies have found that biomass growth is coupled not only with higher N/C and P/C ratios, but also with lower N/P ratios in many heterotrophic organisms, including bacteria^29^.

Table 2 shows the microalgal mean daily productivity, sewage pollutant removal efficiencies and CH_4_ content in biogas under three cultures and various C/N ratios. The behavior of the three selected cultures varied even under identical environmental conditions and media. In both the mono-algae and the algal-fungal culture, biomass grew faster in the C5N1-COD200 treatment than in the other treatments (*p* < 0.05). In the present study, biomass productivity of the algal-fungal culture was higher than the other two cultures. The highest biomass productivity (0.44 g L^−1^ d^−1^, Table 2) for algal-fungal culture was found in C5N1-COD200, although there was no differences among other C/N ratios (*p* < 0.05).

**Table 2:** Mean values ± SD of the removal efficiency of biogas CO_2_ and pollutants removal of different C/N ratio at three different methods of treatments. Values with different superscript letters in the same column for the same method of treatments indicate significant differences at *p* = 0.05 according to Duncan’s multiple range tests.

C/N ratio		Effluent			Upgraded biogas	Biomass productivity
		COD Removal (%)	TN Removal (%)	TP Removal (%)	Concentration of CH_4_ (%,v/v)	Mean daily productivity (g L^−1^ d^−1^)
**Mono-algae culture**						
C2.5N1-COD100	Low COD level	69.34^b^ ± 2.18	69.83^b^ ± 2.18	62.91^c^ ± 3.24	86.33^b^ ± 2.93	0.18^b^ ± 0.02
C5N1-COD200	Medium COD level	72.09^b^ ± 3.26	71.89^a,b^ ± 3.26	69.34^b^ ± 3.11	92.54^a^ ± 4.57	0.24^a^ ± 0.02
C10N1-COD400	High COD level	67.07^b^ ± 3.43	68.45^b^ ± 3.43	63.95^c^ ± 2.96	88.21^a,b^ ± 3.09	0.16^b^ ± 0.01
C10N1-TN20	Low TN level	72.58^b^ ± 2.97	75.16^a^ ± 2.97	70.48^a,b^ ± 3.17	87.95^b^ ± 3.38	0.19^b^ ± 0.02
C5N1-TN40	Medium TN level	77.64^a^ ± 4.35	77.06^a,b^ ± 4.35	73.06^a^ ± 4.35	93.62^a^ ± 3.47	0.25^a^ ± 0.03
C2.5N1-TN80	High TN level	68.75^b^ ± 3.82	75.38^a^ ± 3.82	68.72^b^ ± 2.76	85.47^b^ ± 3.92	0.17^b^ ± 0.01
**Algal-fungal culture**						
C2.5N1-COD100	Low COD level	78.65^a^ ± 4.02	75.08^b^ ± 5.12	75.94^b^ ± 4.09	90.58^ab^ ± 3.46	0.35^b^ ± 0.04
C5N1-COD200	Medium COD level	81.92^a^ ± 4.57	79.35^a^ ± 4.26	79.88^a^ ± 3.87	93.04^a^ ± 2.11	0.44^a^ ± 0.05
C10N1-COD400	High COD level	79.11^a^ ± 2.43	75.54^b^ ± 4.08	77.81^a,b^ ± 4.16	89.37^b^ ± 3.92	0.29^c^ ± 0.02
C10N1-TN20	Low TN level	79.13^a^ ± 3.77	78.42^a^ ± 3.17	78.47^b^ ± 3.57	91.04^a,b^ ± 3.76	0.39^b^ ± 0.03
C5N1-TN40	Medium TN level	80.64^a^ ± 3.92	81.66^a^ ± 6.61	81.52^a^ ± 3.98	93.25^a^ ± 3.84	0.43^a^ ± 0.04
C2.5N1-TN80	High TN level	79.35^a^ ± 5.08	75.15^b^ ± 5.05	74.44^b^ ± 3.19	88.83^b^ ± 4.37	0.31^bc^ ± 0.02
**Algal-bacterial culture**						
C2.5N1-COD100	Low COD level	74.79^b^ ± 3.56	76.95^b^ ± 3.27	69.08^c^ ± 4.02	88.42^ab^ ± 3.58	0.23^ab^ ± 0.03
C5N1-COD200	Medium COD level	77.41^a,b^ ± 4.81	79.48^a^ ± 2.99	75.84^a,b^ ± 2.98	91.54^a^ ± 2.77	0.26^a^ ± 0.02
C10N1-COD400	High COD level	71.60^c^ ± 3.25	75.32^b^ ± 4.81	70.39^c^ ± 4.57	88.53^b^ ± 3.01	0.17^c^ ± 0.02
C10N1-TN20	Low TN level	78.17^a^ ± 5.79	79.83^a^ ± 3.18	74.60^a,b^ ± 3.66	90.39^a,b^ ± 3.54	0.28^a^ ± 0.03
C5N1-TN40	Medium TN level	80.59^a^ ± 3.99	82.28^a^ ± 3.36	77.02^a^ ± 4.09	91.75^a^ ± 2.46	0.24^a^ ± 0.02
C2.5N1-TN80	High TN level	75.02^b^ ± 4.17	75.11^b^ ± 2.56	74.79^b^ ± 3.43	86.19^b^ ± 3.45	0.21^b^ ± 0.02

### Pollutants removal efficiencies

**Figure 1:**
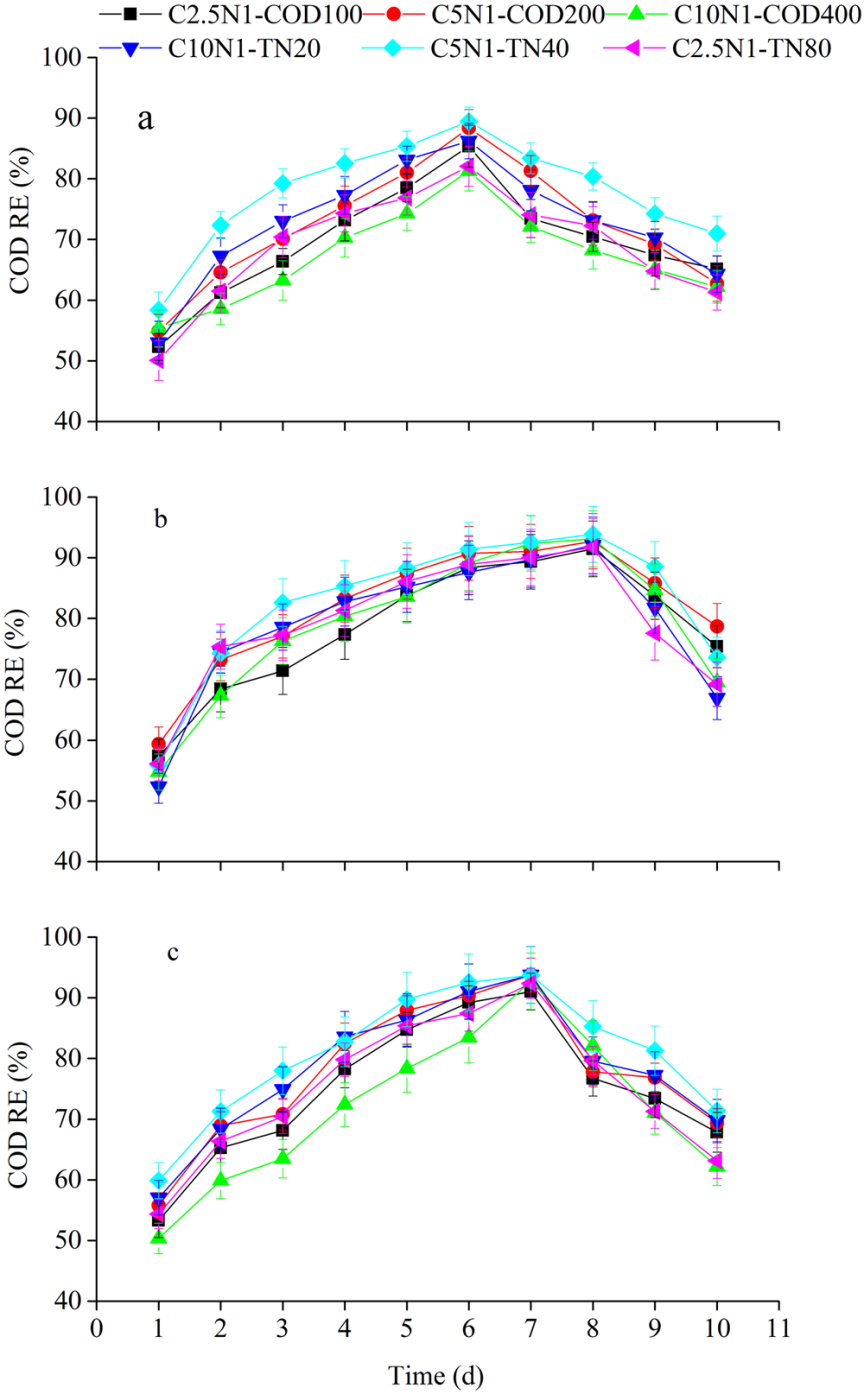
COD removal efficiency over time under various influent C/N ratios for three algae source cultures: (**a**) microalgal monoculture, (**b**) microalgal-fungal co-culture, and (**c**) algal-bacterial co-culture.

**Figure 2:**
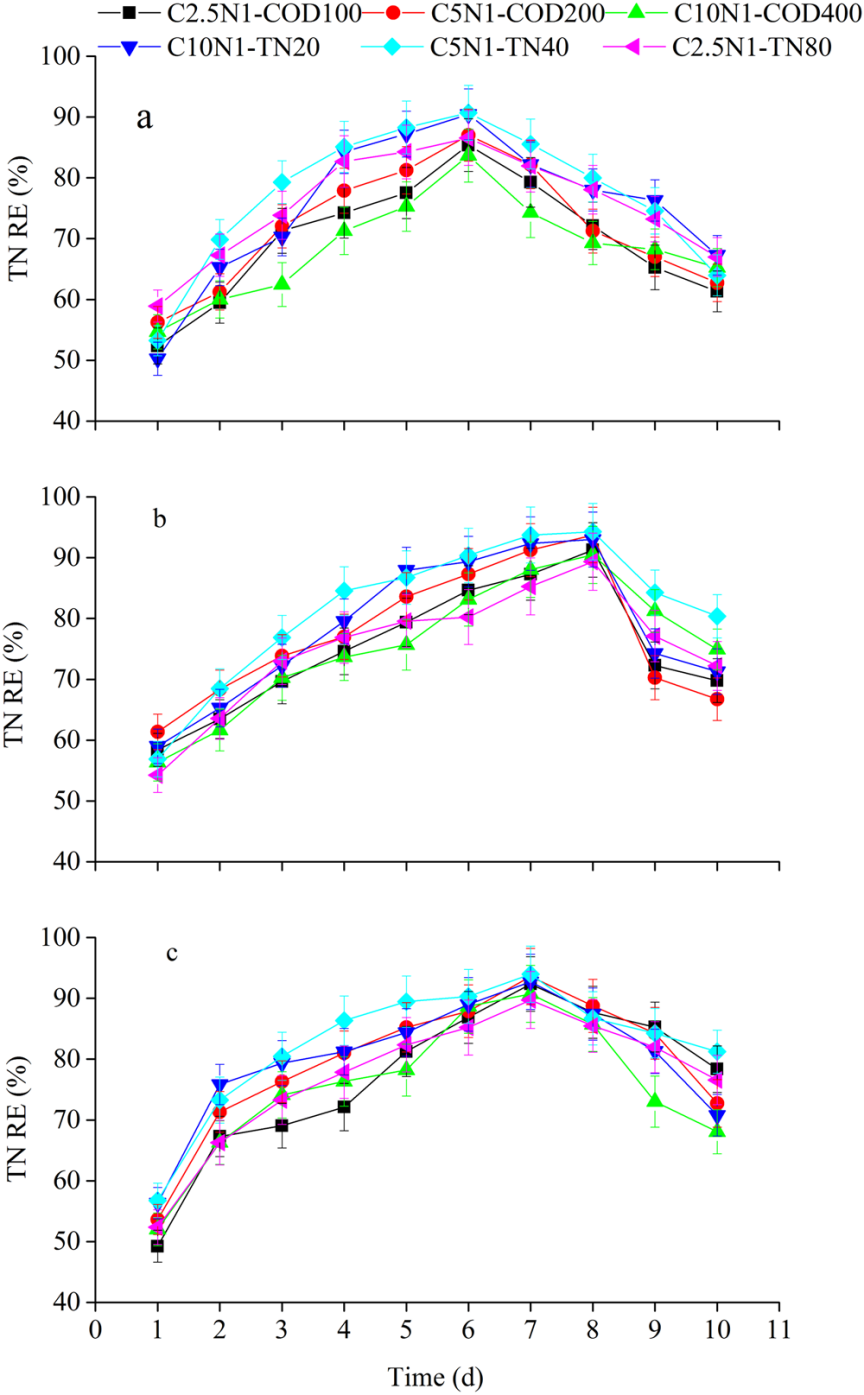
TN removal efficiency over time under various influent C/N ratios for three algae source cultures: (**a**) microalgal monoculture, (**b**) microalgal-fungal co-culture, and (**c**) algal-bacterial co-culture.

**Figure 3:**
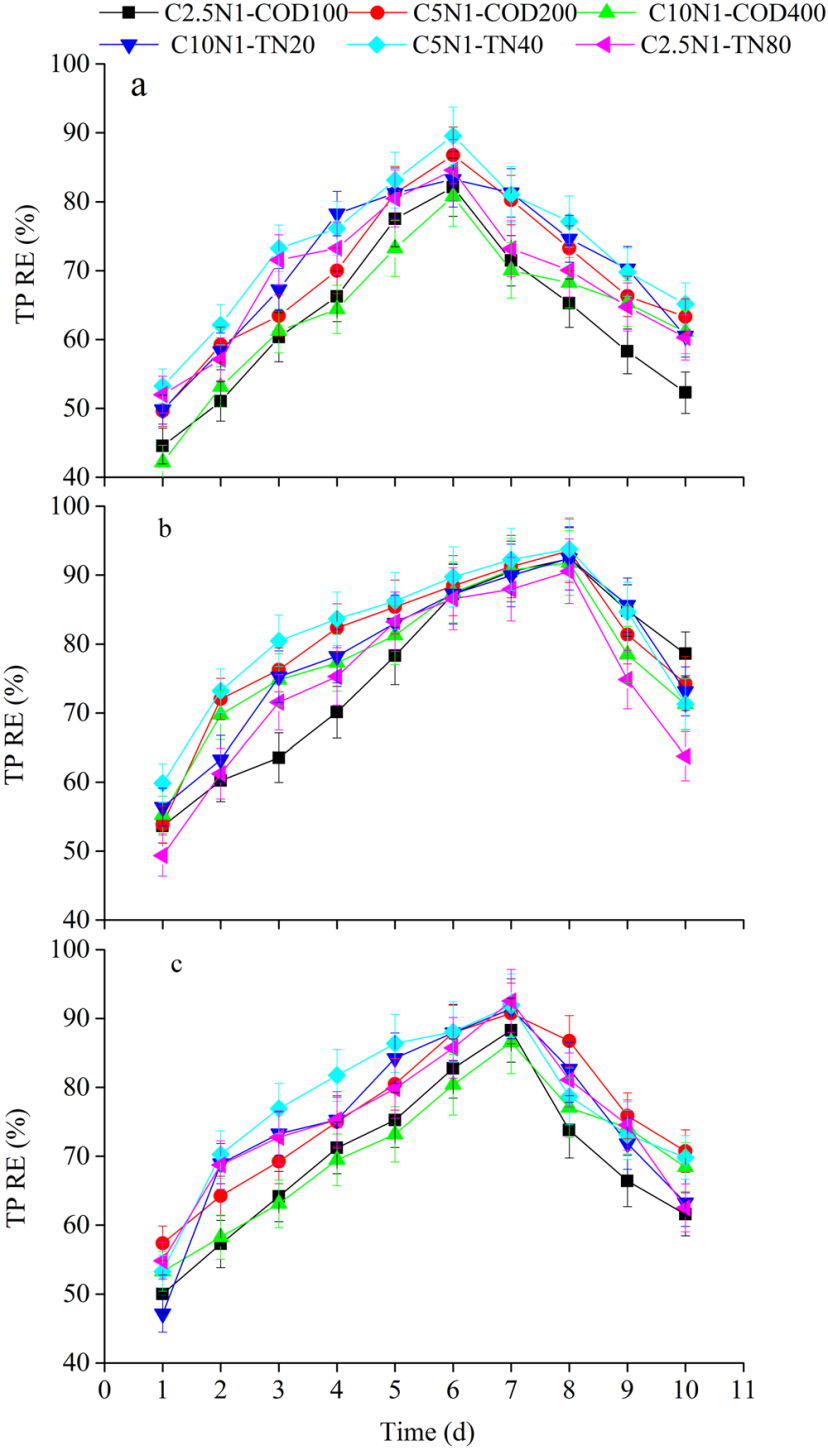
T P removal efficiency over time under various influent C/N ratios for three algae sources cultures: (**a**) microalgal monoculture, (**b**) microalgal-fungal co-culture, and (**c**) algal-bacterial co-culture.

Pollutants uptake by the three selected cultures significantly contributed to COD, TN and TP removal from the wastewater. Figures 1–3 indicated the effects of various influent C/N ratios and culture methods on COD, TN, and TP removal efficiencies using microalgal-based technologies. All the cultures studied efficiently removed pollutants (COD, TN, and TP) from the synthetic domestic sewage. Pollutant removal efficiencies fluctuated for the three algae source culture during the operational period, and the variation tendencies of pollutant removal were similar under different influent C/N ratios. The COD removal efficiencies varied between 50.06% and 89.47% for the microalgal monoculture (Fig. 1a), between 52.36% and 93.87% for the microalgal-fungal co-culture (Fig. 1b), and between 50.35% and 93.78% for the algal-bacterial co-culture (Fig. 1c) under different COD or TN level treatments. The TN removal efficiency variations for the microalgal monoculture ranged from 50.26% to 90.69% (Fig. 2a), for the microalgal-fungal co-culture from 54.25% to 94.28 % (Fig. 2b), and for the algal-bacterial co-culture from 49.23% to 93.97% (Fig. 2c). The TP removal efficiencies for the microalgal monoculture varied between 42.17% and 89.57% (Fig. 3a), for the microalgal-fungal co-culture between 49.36% and 93.77% (Fig. 3b), and for the algal-bacterial co-culture between 47.14% and 92.54% (Fig. 3c). It is noteworthy that after the first day of the assays, pollutant concentrations (in terms of COD, TN, and TP) suddenly dropped. This initial drop implied a COD removal of up to 50.06%, TN removal of up to 49.23%, and TP removal of up to 42.17% in all treatments and was attributed to the adsorption of the microalgae cell walls and subsequent assimilation. The highest pollutant removal efficiencies were observed on day 6 (Figs 1a–3a), day 8 (Figs 1b–3b), and day 7 (Figs 1c–3c) by mono-algae culture, algal-fungal culture, and algal-bacterial culture, respectively. Afterwards, removal efficiencies decreased until the end of the experimental period. This decrease was probably due to the accumulation of metabolic waste, which ultimately led to cell death^20^. Therefore it is suggested that the cultivation times of 6, 8, and 7 days for mono-algae culture, algal-fungal culture, and algal-bacterial culture, respectively, are needed to achieve the highest pollutants removal efficiencies.

In carbon addition treatments, the C5N1-COD200 treatment (69.34%) had a higher TP removal efficiency than the C2.5N1-COD100 (62.91%) and C10N1-COD400 treatments (63.95%) (*p* < 0.05), but no significant difference (*p* > 0.05) was observed in removal efficiencies for COD and TN among C variation treatments by the mono-algae culture. For the algal-fungal culture, the C5N1-COD200 treatment had a higher TN removal efficiency (79.35%) than C2.5N1-COD100 (75.08%) and C10N1-COD400 (75.54%) (*p* < 0.05), but the influent C/N ratios showed no statistically significant effects (*p* > 0.05) on COD removal. For the algal-bacterial culture, the highest TN (79.48%) and TP (69.34%) removal efficiencies were achieved by C5N1-COD200 (*p* < 0.05), and the C10N1-COD400 treatment had a lower COD removal efficiency (71.60%) than the C2.5N1-COD100 (74.79%) and C5N1-COD200 treatments (77.41%) (*p* < 0.05).

Under N addition treatments, the C5N1-TN40 treatment had a higher COD removal efficiency (77.64%) than the C10N1-TN20 (72.58%) and C2.5N1-TN80 treatments (68.75%) (*p* < 0.05), but the N variation treatments (C10N1-TN20, C5N1-TN40, and C2.5N1-TN80) did not significantly affect (*p* > 0.05) the TN removal of the mono-algae culture. For the algal-fungal culture, the C5N1-TN40 treatment showed a significantly higher (*p* < 0.05) TP removal efficiency (81.52%) than those of the C10N1-TN20 (78.47%) and C2.5N1-TN80 treatments (74.44%), and the C2.5N1-TN80 treatment had a lower TN removal efficiency (75.15%) than the C10N1-TN20 (78.42%) and C5N1-TN40 treatments (81.66%) (*p* < 0.05), but no significant difference (*p* > 0.05) was detected in removal efficiencies of COD among different N treatments. However, in the algal-bacterial culture, the C2.5N1-TN80 treatment had significantly lower (*p* <  0.05) COD (75.02%) and TN (75.11%) removal efficiencies than the other treatments.

As shown in Table 3, the interaction of treatment methods and influent C/N ratios as well as the treatment methods significantly influenced pollutants removal efficiencies (*p* < 0.05). Only TN and TP removal efficiency were significantly affected (*p* < 0.05) by influent C/N ratios. This shows that appropriate selection of treatment methods and influent C/N ratios is a simple and effective strategy to increase pollutants removal efficiencies.

**Table 3:** P-values of factors and combined effects of factors for each parameter based on analysis of variance.

Factor	COD RE (%)	TN RE (%)	TP RE (%)	CO_2_ RE (%)
Influent C/N ratios	0.059	0.027*	0.039*	0.067
Treatment methods	0.034*	0.018*	0.044*	0.025*
Influent C/N ratios ×Treatment methods	0.028*	0.032*	0.017*	0.011*

Influent C/N ratios: C variation treatments (C2.5N1-COD100, C5N1-COD200, and C10N1-COD400) and N variation treatments (C10N1-TN20, C5N1-TN40, and C2.5N1-TN80); treatment methods: microalgal monoculture, microalgal-fungal coculture and microalgal-activated sludge coculture (*p < 0.05).

Carbon is a basal element for microalgal growth, contributing to up to about 50% of microalgal biomass^35^. A previous study accessed the carbon mass balance and found that assimilation into biomass was the main carbon removal pathway^20^. Dissolved nitrogen and phosphorus in wastewater could be efficiently removed through continuous microalgal growth. Total N was mainly reduced via microalgal assimilation, as algae cells require nitrogen for protein, nucleic acid, and phospholipid synthesis^9^. Previous work has documented that in wastewater, simple organic nitrogen, including urea and amino acids, can be assimilated by microalgae^15^. Thus, microalgae growth is essential for nitrogen removal via uptake, decay, and sedimentation^36^. On the other hand, partial loss of TN could be attributed to the physical absorption by strain complexes because of their unique structure^37^. In addition, phosphorus is an important nutrient in algal production as a constituent of phospholipids (for cell membranes) and adenosine triphosphate (to supply energy for cell functions), although it constitutes less than 1% of the biomass^38^. In a similar study, Yan *et al*. (2014) obtained a phosphorus removal efficiency of 73.89% for *Chlorella* sp. in biogas slurry^8^.

Carbon dioxide dissolving in sewage will provides a substrate for the microalgal photosynthesis. The dissolution of carbon dioxide promoted the growth of microalgae and therefore played a key role in nitrogen and phosphorus removal. Accordingly, coculture of microalgal and fungi or bacteria promotes the purification of sewage and biogas upgrading^14^. The rich carbon dioxide in the biogas slurry promotes the growth of algal bacteria and increases the biomass of algae bacteria, thus increases the absorption of N and P by algae and improes the sewage purification capacity of algae bacteria.

Several researchers have suggested that denitrification rates in a reactor depend largely upon the amounts of nitrate N and organic carbon as well as on environmental conditions such as pH, temperature, and DO concentration^39^. Here, influent pH levels (7.11–7.54) (Table 1) were within the optimum range for denitrification reported by Paul and Clark (1989)^40^. In practice, nitrogen removal is highly dependent on pollution load levels. In this study, different influent C/N ratios resulted in different denitrification rates, and high TN removal efficiency occurred only if the C/N ratio was 5:1. Consequently, influent C/N ratio plays a crucial role in wastewater treatment^39^. Some scholars have suggested that influent C/N ratio is a domain value, rather than a specific value, because when the experimental influent C/N ratio approaches this value, optimal pollutant removal efficiencies can be achieved^27^. In this research, the balance of carbon and nitrogen nutrient sources in the influent affected the growth of the culture, thereby affecting the nutrient removal efficiencies of the microalgae. On the other hand, the synthetic wastewater used in this research contained carbamide; a high C/N ratio might thus inhibit the removal of ammonia nitrogen due to the competition for DO demand. Therefore, when carbon sources were lacking (C:N = 2.5:1) or nitrogen sources were insufficient (C:N = 10:1), pollutants removal decreased. Considering the combined removal efficiencies for all pollutants, the optimal C/N ratio in this research was 5:1. These results are in agreement with the findings of Yan *et al*.^26^, who reported that influent C/N ratio significantly affected nutrient removal efficiency and that the highest removal effect was found with a medium influent C/N ratio.

Influent C/N ratio also affected effluent phosphorus concentrations, and at C/N 5:1, all treatment systems reached their highest TP removal efficiencies. In the nitrogen addition treatments, the highest average removal efficiencies for the three algae source culture occurred at the C/N ratio of 5:1 (Table 2). These results could be explained by the combined carbon and nitrogen effects for removal of TP. Overall, the results showed that appropriate control of carbon and nitrogen input were necessary to achieve the efficient phosphorus removal.

In the present study, among the three selected cultures, the algal-fungal culture showed the highest pollutants removal efficiencies (COD, TN and TP). The sphere structure of the fungus–algae complex was stable and did not easily break into small pieces (data not shown), which might partially explains the high pollutants removal efficiency. According to the references^14, 41^, when fungi associated with microalgae grew in wastewater, the fungus was pelletized with microalgae cells through bioflocculation and non-bioflocculation. The coagulative mechanism is spore coagulation resulting in accumulation of pellets. The non-coagulative mechanism includes that the spores germinate into hyphae and then intertwines into pellets. In the symbiotic system of algae-fungi/bacteria, on one hand, the extracellular metabolites produced and secreted by the microalgae can be effectively taken up by the surrounding fungi/bacteria for the growth and reproduction. On the other hand, the bacteria in the metabolic process not only can produce the necessary nutrients and growth factors for the growth of microalgae, but also can directly or indirectly regulate the growth environment of microalgae. This forms a mutually beneficial symbiotic relationship^42^. The intergrowth of microalgae and fungi enhances the specific surface area of algae-fungi symbionts and thus the nutrient intake capacity^43^. According to Table 2, coculture of microalgae and fungi resulted in higher nutrient removal as well as CO2 removal except for TN removal. The algal-bacterial culture showed higher TN removal efficiency than algal-fungi culture maybe attribute to the nitrification of bacteria in activated sludge^44^.

### Biogas upgrading

**Figure 4:**
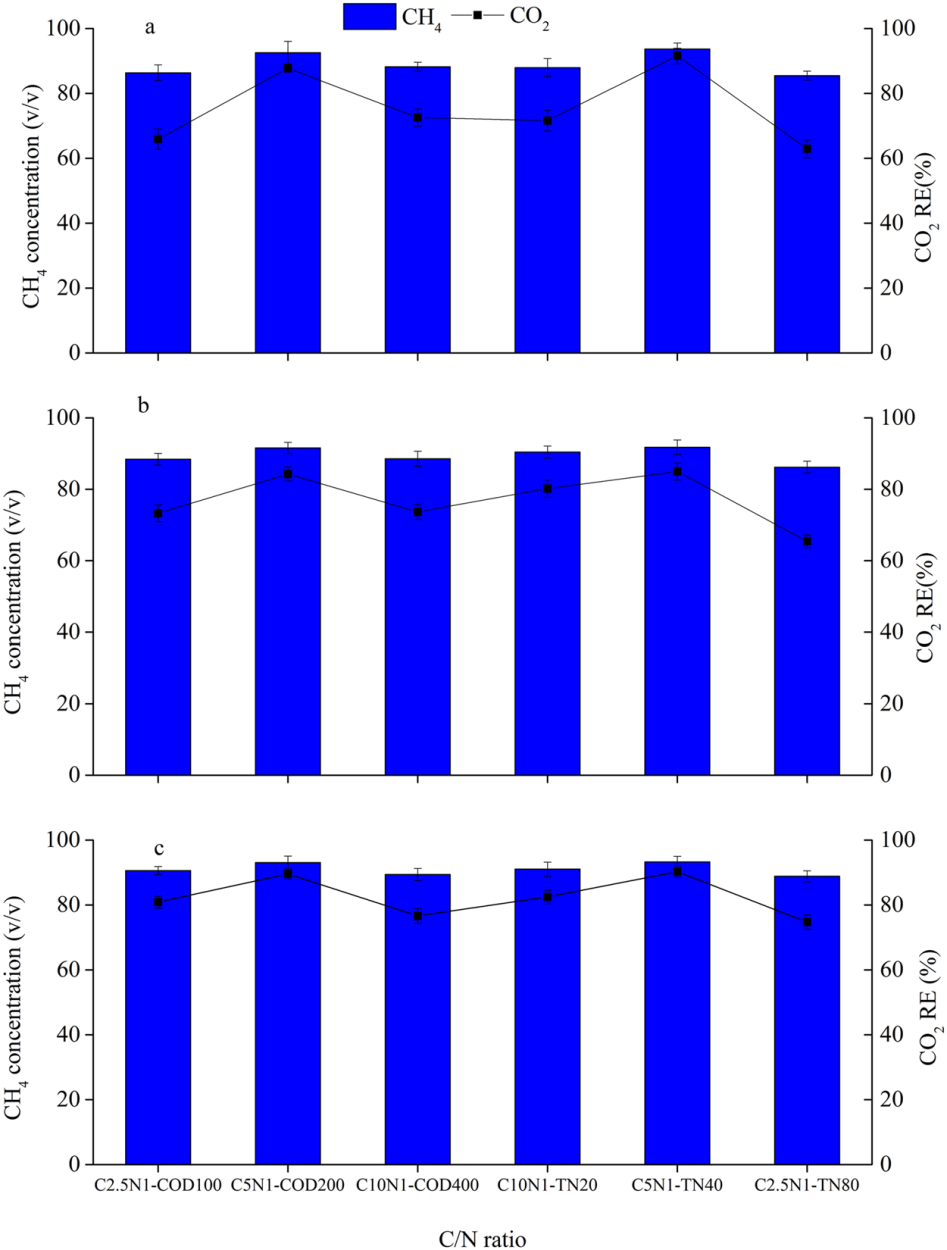
CO_2_ removal efficiencies and final CH_4_ contents (v/v) in biogas under six nutrient concentration levels for three algae source cultures: (**a**) microalgal monoculture, (**b**) microalgal-fungal co-culture, and (**c**) algal-bacterial co-culture.

At the end of the experiment, the CH_4_ contents (v/v) of the biogas and the CO_2_ removal efficiency (%) were investigated to evaluate differences in biogas upgrade with varying influent C/N ratios for the three selected cultures (Fig. 4a–c). The results showed that the tendencies of CH_4_ contents and CO_2_ removal efficiencies were similar to biomass productivities of the three selected culture, as shown in Table 2.

During biogas upgrading, CO_2_ could be effectively removed. In all treatments using algal-bacterial culture, the CO_2_ removal percentage (%) ranged from 74.71% to 90.26%, which was slightly higher than that observed for mono-algae culture (62.89–91.56%) and algal-fungal culture (65.42–84.98%). The overall CO_2_ removal percentage followed similar trends and reached the highest values of 91.56 ± 2.36%, 84.98 ± 2.36%, and 90.26 ± 1.76% in the C5N1-TN40 treatment by mono-algae culture, algal-fungal culture, and algal-bacterial culture, respectively. Under the different C and N variation treatments, no significant differences (*p* > 0.05) in biogas CO_2_ removal efficiencies were found between C5N1-COD200 and C5N1-TN40 among the three microalgal cultures, but their corresponding removal efficiencies were significantly higher (*p* < 0.05) than that in C2.5N1-COD100, C10N1-COD400, C10N1-TN20, and C2.5N1-TN80.

During the experiment, biogas upgrading was affected by the initial pollutants concentrations, and the CH_4_ content (v/v) increased with CO_2_ contents (v/v) decreasing. The differences in the various treatments for the CH_4_ contents (v/v) of the biogas mainly resulted from the variations in the CO_2_ removal, because CH_4_ and CO_2_ contents (v/v) in biogas were significantly negatively correlated. The CH_4_ contents (v/v) enriched by algal-bacterial culture in upgraded biogas in the treatments C2.5N1-COD100, C5N1-COD200, C10N1-COD400, C10N1-TN20, C5N1-TN40, and C2.5N1-TN80 were 90.58 ± 1.25%, 93.04 ± 2.01%, 89.37 ± 1.88%, 91.04 ± 2.16%, 93.25 ± 1.74%, and 88.83 ± 1.69%, respectively;

The CO_2_ in biogas was utilized for algal photosynthesis. As a result, the CH_4_ content in biogas was increased. The CH_4_ content in biogas upgraded by algal-bacterial culture was 88.8%~93.3%, which was higher than those by mono-algae culture and algal-fungal culture. The CH_4_ contents (v/v) enriched through removing CO_2_ by algal-bacterial culture reached the standard of the fuel (CH_4_ > 90%, v/v) in the treatments C5N1-COD200, C10N1-TN20, and C5N1-TN40. Only in C10N1-COD400 and the C2.5N1-TN80, algal-fungal culture, the CH_4_ contents (v/v) did not reach this standard. The highest CH_4_ content (v/v) in the different C and N variation treatments was found in C5N1-TN40, when using the three cultures.

In this research, the effect of biogas upgrading followed similar trends as biomass productivity (Table 2), mainly because approximately half of the microalgal DW was CO_2_-derived carbon^45^. When the microalgae were cultivated in synthetic domestic sewage, CO_2_ was used for microalgal photosynthesis. The microalgae developed a CO_2_-concentrating mechanism to adapt to the changes in the CO_2_ concentration^46, 47^.

### Economic efficiency of the energy consumption

The economic efficiency of energy consumption on synthetic domestic sewage purification and biogas upgrading showed a similar variation (Table 4). The highest economic efficiency of pollutants and CO_2_ removal with different culture methods can be found at the C/N ratio of 5:1 with the medium TN level. This finding is corresponded to the variation of the microalgal biomass productivity. To be specific, algal-fungal culture achieved the highest economic efficiency of COD and TP removal; algal-bacterial culture achieved the highest economic efficiency of TN and CO_2_ removal. The ANOVA showed that there was no significant difference in COD, TN and TP removal rates between algal-fungi and algal-bacteria cultures (*p* > 0.05), and there was no significant difference on energy efficiency CO_2_ removal by the three cultures (p > 0.05). For these two cocultivations, the energy efficiencies of pollutant removal were higher, but energy efficiencies of CO_2_ removal were lower of than that of mono-algae culture. Microalgae will grow well in the influent C/N ratio of 5:1 and improve the sewage purification, as a result, the economic efficiency of energy consumption are increased^13^. For algal-bacteria culture, due to nitrification and denitrification of activated sludge, the economic efficiency of nitrogen removal achieve higher than algal-fungal culture^39, 44^.

**Table 4:** The economic efficiency of energy consumption on synthetic domestic sewage purification and biogas upgrading at different C/N ratio with three different culture methods.

C/N ratio		Economic efficiency (USD^−1^)			
		COD	TN	TP	CO_2_
**Mono-algae culture**					
C2.5N1-COD100	Low COD level	37.34^b,c^ ± 2.28	37.42^b,c^ ± 2.37	27.28^c^ ± 1.74	29.58^c^ ± 1.94
C5N1-COD200	Medium COD level	39.81^b^ ± 2.74	39.09^b^ ± 2.58	36.44^b^ ± 2.06	37.63^a^ ± 3.18
C10N1-COD400	High COD level	36.16^c^ ± 1.97	36.77^c^ ± 2.61	28.36^c^ ± 1.93	35.44^b^ ± 2.86
C10N1-TN20	Low TN level	39.96^b^ ± 2.45	42.33^a^ ± 2.53	37.24^b^ ± 2.32	33.89^b^ ± 3.05
C5N1-TN40	Medium TN level	43.27^a^ ± 3.18	43.07^a^ ± 3.18	40.31^a^ ± 2.17	38.76^a^ ± 2.89
C2.5N1-TN80	High TN level	35.83^c^ ± 1.84	42.58^a^ ± 2.72	35.57^b^ ± 2.09	28.07^c^ ± 2.77
**Algal-fungal culture**					
C2.5N1-COD100	Low COD level	44.28^b^ ± 3.22	42.26^b^ ± 2.34	43.19^b^ ± 2.75	31.42^c^ ± 2.35
C5N1-COD200	Medium COD level	46.16^a^ ± 2.83	45.78^a^ ± 2.57	45.98^a^ ± 2.37	38.17^a^ ± 2.49
C10N1-COD400	High COD level	45.07^ab^ ± 2.71	42.63^b^ ± 2.28	43.52^b^ ± 2.63	30.61^c^ ± 2.78
C10N1-TN20	Low TN level	45.19^a,b^ ± 3.05	43.92^a,b^ ± 3.08	44.13^a,b^ ± 2.79	37.94^a^ ± 3.37
C5N1-TN40	Medium TN level	45.75^a,b^ ± 3.29	45.99^a^ ± 3.11	46.37^a^ ± 3.07	38.23^a^ ± 2.34
C2.5N1-TN80	High TN level	45.41^ab^ ± 2.98	42.18^b^ ± 2.62	42.23^b^ ± 2.66	34.73^b^ ± 3.39
**Algal-bacterial culture**					
C2.5N1-COD100	Low COD level	41.62^b,c^ ± 2.65	42.34^b^ ± 2.37	36.05^c^ ± 2.17	35.59^b^ ± 2.68
C5N1-COD200	Medium COD level	42.87^b^ ± 3.01	45.58^a^ ± 2.68	42.51^a^ ± 3.79	38.21^a^ ± 3.27
C10N1-COD400	High COD level	39.25^c^ ± 2.42	42.41^b^ ± 2.26	36.87^c^ ± 2.96	35.79^b^ ± 2.96
C10N1-TN20	Low TN level	43.83^a,b^ ± 2.79	45.72^a^ ± 2.89	40.94^b^ ± 2.34	37.32^a^ ± 3.11
C5N1-TN40	Medium TN level	45.32^a^ ± 2.61	46.65^a^ ± 3.42	42.98^a^ ± 3.83	38.63^a^ ± 3.09
C2.5N1-TN80	High TN level	42.12^b^ ± 2.83	42.52^b^ ± 2.63	41.22^ab^ ± 2.62	28.85^c^ ± 2.83

Values with different superscript letters in the same column for the same method of treatments indicate significant differences at *p* = 0.05 according to Duncan’s multiple range tests.

## Conclusion

Both of the microalgal culture methods and C/N ratios had significant effects on of synthetic domestic sewage purification and biogas upgrading. The medium level of C/N ratio showed higher pollutants and CO_2_ removal efficiencies than low and high C/N ratios. Co-culture of *C. vulgaris* with *G. lucidum* or activated sludge in photobioreactors was more effective than mono-cultivation on removing sewage pollutants and CO_2_ in biogas simultaneously according to the data in this study. Coculture of microalgae with fungi was the suitable treatment technology for wastewater purification and biogas upgrading under the C/N ratio of 5:1.

## Methods

### Algae sources and culture conditions

The *C. vulgaris* (FACHB-31) was used for sewage treatment and biogas upgrading based on its high biogas tolerance and rapid growth in high-strength wastewater^48^. The strain was purchased from the FACHB-Collection, Institute of Hydrobiology, Chinese Academy of Sciences. The BG-11 culture media was prepared for growing the microalgal culture^49^, with initial pH adjusted to 6.9. The culture conditions were as follows: the wavelength spectrum and photosynthetic photon flux density (PPFD) of cool-white LED light were 360~720 nm and 200 μmol m^−2^ s^−1^, respectively. The temperature and light period were 25 ± 0.5 °C and 12 h light-12 h dark, respectively. The cultures were intermittently shaken three times a day (8:00 a.m., 2:00 p.m., and 8:00 p.m.).

### Algal-fungal culture conditions

Based on the preliminary experiment results, we found that *Ganoderma lucidum* (*G. lucidum*, 5.765) achieved high growth rate and high performance of pelletization with *C. vulgaris*. Therefore, the *Ganoderma lucidum* stain obtained from China General Microbiological Culture Collection Center was selected for this study. An inoculum was prepared by inoculating 100 mL of a synthetic medium with 25 mycelial discs. The composition of synthetic medium was as follows: glucose, 10 g L^−1^; NH_4_NO_3_, 2.0 g L^−1^; K_2_HPO_4_, 1.0 g L^−1^; NaH_2_PO_4_·H_2_O, 0.4 g L^−1^; MgSO_4_·7H_2_O, 0.5 g L^−1^; yeast extract, 2.0 g L^−1^; pH 6.5. The prepared inoculum was then incubated at 25 ± 1 °C on a rotary shaker at 160 rpm for 7 d. The obtained biomass was washed with sterile distilled water and homogenized with 100 mL of sterile distilled water in a laboratory blender. Subsequently, the obtained cultures were used for the co-cultivation of microalgal cells.

Each 100 mL Microalgal suspension (158.37 ± 14.26 mg L^−1^) was mixed with 5 mL *G. lucidum* suspension (82 ± 8 mg L^−1^) for pelletization. The fungal-algal mixtures were shaken at 160 rpm for 7d under constant PPFD (200 μmol m^−2^ s^−1^) at 25 °C. All experiments were performed in triplicate.

### Algal-bacterial Culture conditions

The synthetic domestic sewage was inoculated with 1L cultured microalgal strain and 200 mL activated sludge. The total suspended solid (TSS) of microalgae and activated sludge were 0.75 g TSS L^−1^and 4.14 g TSS, respectively. The activated sludge came from a wastewater treatment plant in Jiaxing, Zhejiang, China. The light intensity and temperature were the same as the algal-fungi culture^20^.

### Photobioreactor

**Figure 5:**
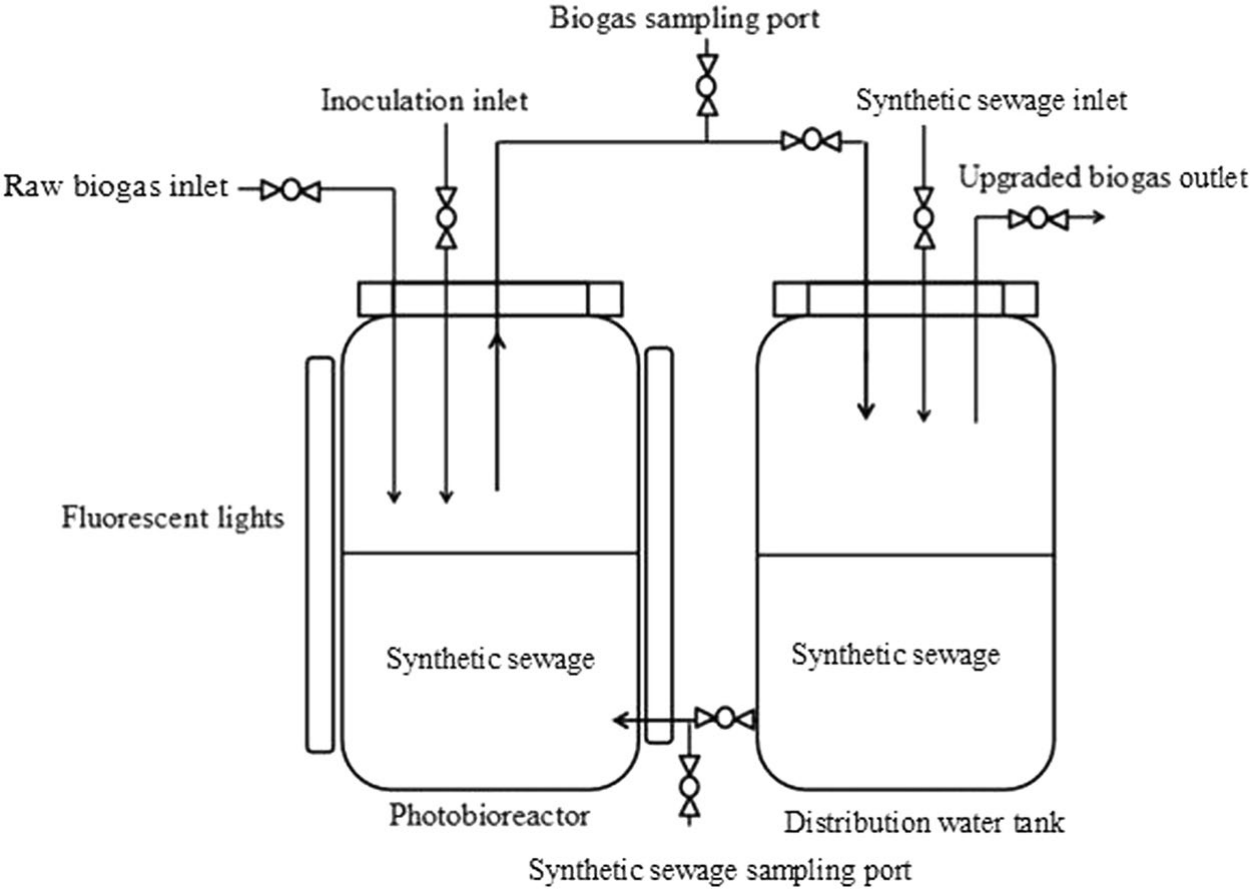
Scheme of the photobioreactor experimental setup.

Two interconnected 16.8 L (individual) glass cylinder blocks with a height of 0.6 m and a diameter of 0.2 m were used as a photobioreactor (Fig. 5). The reactors were hermetically sealed with rubber stoppers. The sampling outlet consisted of a plug and rubber gasket. The synthetic domestic sewage was pumped from the right to the left cylinder block of the photobioreactor in one time. The raw biogas was blown into the photoreactors from the raw biogas inlet until the air in the headspace was expelled.

### Synthetic domestic sewage and biogas

To facilitate comparison with similar experiments, this study used the synthetic domestic sewage, a modification of the OECD standard sewage^44^. The concentrations of TN and COD were adjusted, whereas the TP was not. The experiment was divided into two groups. In group 1, low (100 mg L^−1^), medium (200 mg L^−1^), and high (400 mg L^−1^) levels of COD (fixed TN/TP levels at medium strength) were designated as C2.5N1-COD100, C5N1-COD200, and C10N1-COD400. In group 2, low (20 mg L^−1^), medium (40 mg L^−1^), and high levels (80 mg L^−1^) of N (fixed COD/TP levels at medium strength) were designated as C2.5N1-TN80, C5N1-TN40, C10N1-TN20^50^. Medium for group 1 was prepared by mixing the following components: 100, 200, and 400 g m^−3^ glucose, respectively; 80 g m^−3^ carbamide; 15 g m^−3^ NaH_2_PO_4_; 1.5 g m^−3^ KH_2_PO_4_; 4 g m^−3^ CaCl_2_; and 2 g m^−3^ MgSO_4_. Medium for group 2 was: 200 g m^−3^ glucose; 40, 80, and 160 g m^−3^ carbamide, respectively; 15 g m^−3^ NaH_2_PO_4_; 1.5 g m^−3^ KH_2_PO_4_; 4 g m^−3^ CaCl_2_; and 2 g m^−3^MgSO_4_^51^. Table 5 shows the characteristics of the synthetic domestic sewage.

**Table 5:** Parameters of influent synthetic sewage and crude biogas in the photobioreactor.

C/N ratio		Influent concentration (mg L^−1^)		
		COD	TN	TP
**Mono-algae culture**				
C2.5N1-COD100	Low COD level	102.16 ± 3.21	43.12 ± 3.54	5.21 ± 0.42
C5N1-COD200	Medium COD level	203.77 ± 6.19	41.08 ± 2.75	5.39 ± 0.57
C10N1-COD400	High COD level	408.35 ± 8.67	44.63 ± 3.18	5.11 ± 0.74
C10N1-TN20	Low TN level	202.49 ± 5.11	20.42 ± 2.06	5.33 ± 0.62
C5N1-TN40	Medium TN level	205.18 ± 4.09	43.71 ± 2.83	5.46 ± 0.83
C2.5N1-TN80	High TN level	206.03 ± 7.16	82.75 ± 4.35	5.08 ± 0.49
**Algal-fungal culture**				
C2.5N1-COD100	Low COD level	103.83 ± 4.28	42.07 ± 2.98	5.41 ± 0.72
C5N1-COD200	Medium COD level	204.39 ± 5.93	44.24 ± 3.04	5.22 ± 0.63
C10N1-COD400	High COD level	403.58 ± 8.47	42.35 ± 3.76	5.35 ± 0.57
C10N1-TN20	Low TN level	206.34 ± 7.53	22.31 ± 2.53	5.05 ± 0.66
C5N1-TN40	Medium TN level	208.37 ± 8.95	41.64 ± 2.32	5.14 ± 0.58
C2.5N1-TN80	High TN level	203.67 ± 7.02	84.09 ± 3.84	5.23 ± 0.67
**Algal-bacterial culture**				
C2.5N1-COD100	Low COD level	106.25 ± 3.89	40.86 ± 3.21	5.39 ± 0.84
C5N1-COD200	Medium COD level	208.02 ± 5.92	42.24 ± 2.95	5.04 ± 0.62
C10N1-COD400	High COD level	407.11 ± 7.81	43.31 ± 3.76	5.28 ± 0.25
C10N1-TN20	Low TN level	205.27 ± 6.47	22.26 ± 2.99	5.19 ± 0.57
C5N1-TN40	Medium TN level	208.81 ± 8.43	41.06 ± 4.33	5.23 ± 0.65
C2.5N1-TN80	High TN level	201.34 ± 7.29	84.32 ± 3.27	5.37 ± 0.53

Biogas was obtained from a farm biogas plant in JiaYuan Green Meadow. Prior to the experiments, the biogas was pretreated via chemical absorption to reduce the H_2_S content less than 0.005% (v/v). The raw biogas mainly consisted of CH_4_ (67.59 ± 4.13%, v/v) and CO_2_ (28.43 ± 3.04%, v/v), trace amounts of other components included H_2_O (3.54 ± 0.27%, v/v), O_2_ (0.47 ± 0.03%, v/v), and H_2_S (<0.005%, v/v).

### Experimental procedure

The photobioreactor was filled with 14 L of raw biogas and 2.8 L of synthetic domestic sewage and illuminated on 200 μmol m^−2^ s^−1^ by six fluorescent lamps arranged in a circular configuration (20 W, 110 V) around the left cylinder block (Fig. 1). The initial dry weight (DW) of the three selected culture was about 90.21 ± 6.39 mg L^−1^ in all samples to obtain similar initial biomass concentrations. The temperature and light/dark cycle of microalgal for wastewater treatment was the same as the microalgal mono-cultivation. Under different influent C/N ratios, growth rate and pollutants removal efficiencies of the three algal source cultures and their roles in biogas upgrading were determined. Gas samples (100 mL) were drawn daily at the sampling outlet to monitor CO_2_ and CH_4_ concentrations. By using a 15-mL syringe, the synthetic domestic sewage was sampled daily at 8:00 a.m. through the sampling port of the photobioreactor, to monitor the COD, TN, and TP concentrations.

All treatments were performed in triplicate. The cultures were sampled and analyzed daily at 8:00 a.m. and mean values were calculated for each time and culture.

### Sampling and analyses

The DW of the three algal source cultures was measured according to the reference^19^. Daily biomass productivity (*P*, g L^−1^ d^−1^) was calculated using Equation (1):

1 $$P=({D}_{i}-{D}_{0})/({t}_{i}-{t}_{0})$$

where *D*_i_ is the biomass concentration (g L^−1^) at time *t*_i_ (d) and *D*_0_ is the initial biomass concentration (g L^−1^) at t_0_ (d).

The culture filtrates were analyzed for COD, TN, and TP concentrations by using standard methods^52^. The pH value and dissolved oxygen (DO) was measured using a pH (Orion 250 Aplus ORP Field Kit, USA) and oxygen probes (Model 862 Aplus, USA). Pollutants (COD, TN, and TP) removal efficiency was calculated as follows:

2 $${\rm{R}}=(1-{{\rm{C}}}_{{\rm{i}}}/{{\rm{C}}}_{0})$$

where R is the pollutant removal efficiency (%), C_0_ and C_i_ are the pollutant concentrations in the initial synthetic domestic sewage and in the filtrates of the cultures (mg L^−1^), respectively.

The contents (v/v) of CO_2_, H_2_S, CH_4_, and O_2_ in the biogas were measured using a circulating gas analyzer^50^.

The economic efficiency of the energy consumption for nutrient removal in sewage and CO_2_ removal in biogas were calculated by Eq. (3)

3 $${\rm{E}}=\frac{{\rm{R}}}{{\rm{KTP}}}$$

where E is the energy consumption for nutrient removal in sewage and CO_2_ removal in biogas, USD^−1^; R is the removal efficiency in Eq. (2), %; k stands for the electric power charge per unit of energy consumption, USD kw^−1^h^−1^; T is the illumination time according to photoperiod, h; P is the LED electrical power consumption, W. The electric power charge per unit of energy consumption k is around 0.08826 USD kw^−1^h^−1^ in local after conversion^13^.

### Statistical analyses

All statistical analyses were performed using the package SPSS (SPSS 2013). A one-way analysis of variance (ANOVA) was used to test the statistical differences of the 6 C/N ratios for the same microalgae cultures. Duncan’s multiple range tests was employed to further test for significant differences among the treatments with different C/N ratios. A two-way ANOVA was used to test for differences among the effects of influent C/N ratios, algae sources, and the interaction between any two of these factors on treatment performance. The threshold for statistical significance was set at *p* = 0.05. Error bars in the figures showed the standard deviation with n = 3.

## References

[1] Wang X, Zhang W, Huang Y, Li S (2004). Modeling and simulation of point-non-point source effluent trading in Taihu Lake area: perspective of non-point sources control in China. Science of the Total Environment.

[2] Zhou X (2012). Enhancing nitrogen removal in an Orbal oxidation ditch by optimization of oxygen supply: practice in a full-scale municipal wastewater treatment plant. Bioprocess and Biosystems Engineering.

[3] Ding P, Chu L, Wang J (2016). Biological treatment of actual petrochemical wastewater using anaerobic/anoxic/oxic process and the microbial diversity analysis. Applied Microbiology & Biotechnology.

[4] Mannina G, Capodici M, Cosenza A, Trapani DD (2016). Carbon and nutrient biological removal in a University of Cape Town membrane bioreactor: Analysis of a pilot plant operated under two different C/N ratios. Chemical Engineering Journal.

[5] Lu YZ, Wang HF, Kotsopoulos TA, Zeng RJ (2016). Advanced phosphorus recovery using a novel SBR system with granular sludge in simultaneous nitrification, denitrification and phosphorus removal process. Applied Microbiology and Biotechnology.

[6] Yang CF, Ding ZY, Zhang KC (2008). Growth of *Chlorella pyrenoidosa* in wastewater from cassava ethanol fermentation. World Journal of Microbiology and Biotechnology.

[7] Ryckebosch E, Drouillon M, Vervaeren H (2011). Techniques for transformation of biogas to biomethane. Biomass & Bioenergy.

[8] Yan C, Zheng Z (2014). Performance of mixed LED light wavelengths on biogas upgrade and biogas fluid removal by microalga *Chlorella sp*. Applied Energy.

[9] Kumar M, Miao Z, Wyatt S (2010). Influence of nutrient loads, feeding frequency and inoculum source on growth of *Chlorella vulgaris* in digested piggery effluent culture medium. Bioresource Technology.

[10] Sandefur HN, Matlock MD, Costello TA (2011). Seasonal productivity of a periphytic algal community for biofuel feedstock generation and nutrient treatment. Ecological Engineering.

[11] Chan Y (2010). Selection of microalgae for lipid production under high levels carbon dioxide. Bioresource Technology.

[12] Zhang Y, Bao K, Wang J, Zhao Y, Hu C (2017). Performance of mixed LED light wavelengths on nutrient removal and biogas upgrading by different microalgal–based treatment technologies. Energy.

[13] Cao W, Wang X, Sun S, Hu C, Y. Z (2017). Simultaneously upgrading biogas and purifying biogas slurry using cocultivation of Chlorella vulgaris and three different fungi under various mixed light wavelength and photoperiods. Bioresource Technology.

[14] Muradov N (2015). Fungal-assisted algal flocculation: application in wastewater treatment and biofuel production. Biotechnology for Biofuels.

[15] Su Y, Mennerich A, Urban B (2011). Municipal wastewater treatment and biomass accumulation with a wastewater-born and settleable algal-bacterial culture. Water Research.

[16] Zoller S, Lutzoni F (2003). Slow algae, fast fungi: exceptionally high nucleotide substitution rate differences between lichenized fungi Omphalina and their symbiotic green algae. Coccomyxa. Molecular Phylogenetics & Evolution.

[17] Deng L, Hägg MB (2010). Techno-economic evaluation of biogas upgrading process using CO_2_ facilitated transport membrane. International Journal of Greenhouse Gas Control.

[18] Sun Q (2015). Selection of appropriate biogas upgrading technology-a review of biogas cleaning, upgrading and utilisation. Renewable & Sustainable Energy Reviews.

[19] Zhang Y, Bao K, Wang J, Zhao Y, Hu C (2017). Performance of mixed LED light wavelengths on nutrient removal and biogas upgrading by different microalgal-based treatment technologies. Energy.

[20] Serejo ML (2015). Influence of biogas flow rate on biomass composition during the optimization of biogas upgrading in microalgal-bacterial processes. Environmental Science & Technology.

[21] Hwang JH, Church J, Lee SJ, Park J, Lee WH (2016). Use of Microalgae for Advanced Wastewater Treatment and Sustainable Bioenergy Generation. Environmental Engineering Science.

[22] Wang X, Bao K, Cao W, Zhao Y, Hu CW (2017). Screening of microalgae for integral biogas slurry nutrient removal and biogas upgrading by different microalgae cultivation technology. Scientific Reports.

[23] Samorì G, Samorì C, Guerrini F, Pistocchi R (2013). Growth and nitrogen removal capacity of Desmodesmus communis and of a natural microalgae consortium in a batch culture system in view of urban wastewater treatment: Part I. Water Research.

[24] García D (2017). Enhanced carbon, nitrogen and phosphorus removal from domestic wastewater in a novel anoxic-aerobic photobioreactor coupled with biogas upgrading. Chemical Engineering Journal.

[25] Muradov N (2015). Fungal-assisted algal flocculation: application in wastewater treatment and biofuel production. Biotechnol. Biofuels.

[26] Yan C (2012). Effects of influent C/N ratios on CO_2_ and CH_4_ emissions from vertical subsurface flow constructed wetlands treating synthetic municipal wastewater. Journal of Hazardous Materials.

[27] Zhao YJ, Liu B, Zhang WG, Ouyang Y, An SQ (2010). Performance of pilot-scale vertical-flow constructed wetlands in responding to variation in influent C/N ratios of simulated urban sewage. Bioresource Technology.

[28] Xu B, Cheng P, Yan C, Pei H, Hu W (2013). The effect of varying LED light sources and influent carbon/nitrogen ratios on treatment of synthetic sanitary sewage using Chlorella vulgaris. World Journal of Microbiology and Biotechnology.

[29] Makino W, Cotner JB, Sterner RW, Elser JJ (2003). Are bacteria more like plants or animals? Growth rate and resource dependence of bacterial C:N:P stoichiometry. Functional Ecology.

[30] Pittman JK, Dean AP, Osundeko O (2011). The potential of sustainable algal biofuel production using wastewater resources. Bioresource Technology.

[31] Papazi A, Makridis P, Divanach P, Kotzabasis K (2008). Bioenergetic changes in the microalgal photosynthetic apparatus by extremely high CO_2_ concentrations induce an intense biomass production. Physiologia Plantarum.

[32] de Godos I, Blanco S, García-Encina PA, Becares E, Muñoz R (2010). Influence of flue gas sparging on the performance of high rate algae ponds treating agro-industrial wastewaters. Journal of Hazardous Materials.

[33] Carvalho AP, Meireles LA, Malcata FX (2006). Microalgal reactors: a review of enclosed system designs and performances. Biotechnology Progress.

[34] Douskova I (2009). Simultaneous flue gas bioremediation and reduction of microalgal biomass production costs. Applied Microbiology and Biotechnology.

[35] Singh, M. & Das, K. C. In *Algal Biorefineries: Volume 1: Cultivation of* Cells *and Products* (eds Rakesh Bajpai, Aleš Prokop, & Mark Zappi) 69–82 (Springer Netherlands, 2014).

[36] Li H (2016). Growth responses of Ulva prolifera to inorganic and organic nutrients: Implications for macroalgal blooms in the southern Yellow Sea, China. Scientific Reports.

[37] Li SL (2011). Isolation, identification and characterization of oleaginous fungi from the soil of Qinghai Plateau that utilize D-xylose. African Journal of Microbiology Research.

[38] Rittenberg SC (1969). The Roles of Exogenous Organic Matter in the Physiology of Chemolithotrophic Bacteria. Advances in Microbial Physiology.

[39] Xia S, Li J, Wang R (2008). Nitrogen removal performance and microbial community structure dynamics response to carbon nitrogen ratio in a compact suspended carrier biofilm reactor. Ecological Engineering.

[40] Paul, E. A. & Clark, F. E. In *Soil Microbiology & Biochemistry* Vol. 51 xi–xii (Academic Press, 1989).

[41] Gultom SO, Hu B (2013). Review of microalgae harvesting via co-Pelletization with filamentous fungus. Energies.

[42] Ferrier M, Martin JL, Rooney-Varga JN (2002). Stimulation of Alexandrium fundyense growth by bacterial assemblages from the Bay of Fundy. Journal of Applied Microbiology.

[43] Zhang J, Hu B (2012). A novel method to harvest microalgae via co-culture of filamentous fungi to form cell pellets. Bioresource Technology.

[44] OECD. Test No. 303: Simulation Test ‐ Aerobic Sewage Treatment A: Activated Sludge Units; B: Biofilms. *Oecd Guidelines for the Testing of Chemicals* **1**, 1–50 (2006).

[45] Chisti Y (2008). Biodiesel from microalgae beats bioethanol. Trends in Biotechnology.

[46] Sun S (2016). Performance of CO_2_ concentrations on nutrient removal and biogas upgrading by integrating microalgal strains cultivation with activated sludge. Energy.

[47] Mudimu O, Rybalka N, Bauersachs T, Friedl T, Schulz R (2015). Influence of Different CO_2_ Concentrations on Microalgae Growth, α-Tocopherol Content and Fatty Acid Composition. Geomicrobiology.

[48] Li B (2013). Purifying biogas slurry and upgrading biogas by *Chlorella vulgaris*. Chinese Journal of Environmental Engineering.

[49] Rippka R, Deruelles J, Waterbury JB, Herdman M, Stanier RY (1979). Generic Assignments, Strain Histories and Properties of Pure Cultures of Cyanobacteria. Microbiology.

[50] Yan C, Zheng Z (2013). Performance of photoperiod and light intensity on biogas upgrade and biogas effluent nutrient reduction by the microalgae Chlorella sp. Bioresource Technology.

[51] Yan C, Zhang L, Luo X, Zheng Z (2013). Effects of various LED light wavelengths and intensities on the performance of purifying synthetic domestic sewage by microalgae at different influent C/N ratios. Ecological Engineering.

[52] Author. *Standard Methods for the Examination of Water and Wastewater, 21st Edition*. Edition edn,American Public Health Association (2006).

